# Evaluation and Comparison of Traditional Plaster and Fiberglass Casts with 3D-Printed PLA and PLA–CaCO_3_ Composite Splints for Bone-Fracture Management

**DOI:** 10.3390/polym14173571

**Published:** 2022-08-30

**Authors:** Ádám Tibor Schlégl, Roland Told, Kinga Kardos, András Szőke, Zoltan Ujfalusi, Péter Maróti

**Affiliations:** 1 Medical Skills Education and Innovation Centre, Medcal School, University of Pécs, Szigeti Street 12, H-7624 Pécs, Hungary; 2 Department of Orthopaedics, Medical School, University of Pécs, Akác Street 1, H-7632 Pécs, Hungary; 3 3D Printing and Visualization Centre, University of Pécs, Boszorkány Road 2, H-7624 Pécs, Hungary; 4 Department of Biophysics, Medical School, University of Pécs, Szigeti Street 12, H-7624 Pecs, Hungary

**Keywords:** casts, medical device, mechanical test, structural analysis, polymer, gypsum, fracture, fracture conservative treatment, PLA, composite, calcium carbonate

## Abstract

Bone fractures pose a serious challenge for the healthcare system worldwide. A total of 17.5% of these fractures occur in the distal radius. Traditional cast materials commonly used for treatment have certain disadvantages, including a lack of mechanical and water resistance, poor hygiene, and odors. Three-dimensional printing is a dynamically developing technology which can potentially replace the traditional casts. The aim of the study was to examine and compare the traditional materials (plaster cast and fiberglass cast) with Polylactic Acid (PLA) and PLA–CaCO_3_ composite materials printed using Fused Filament Fabrication (FFF) technology and to produce a usable cast of each material. The materials were characterized by tensile, flexural, Charpy impact, Shore D hardness, flexural fatigue, and variable load cyclic tests, as well as an absorbed water test. In addition, cost-effectiveness was evaluated and compared. The measured values for tensile strength and flexural strength decreased with the increase in CaCO_3_ concentration. In the fatigue tests, the plaster cast and the fiberglass cast did not show normal fatigue curves; only the 3D-printed materials did so. Variable load cyclic tests showed that traditional casts cannot hold the same load at the same deflection after a higher load has been used. During these tests, the plaster cast had the biggest relative change (−79.7%), compared with −4.8 % for the 3D-printed materials. The results clearly showed that 3D-printed materials perform better in both static and dynamic mechanical tests; therefore, 3D printing could be a good alternative to customized splints and casts in the near future.

## 1. Introduction

Bone fractures present a major challenge to the healthcare system and also a significant financial burden for society and the patient. Osteoporosis-associated fractures alone cost AUD 2346.08 million in 2017 in Australia [1]. They are a relatively common condition, although we do not know their exact incidence. The available data vary between 9.0 and 22.8 fractures/1000 people/year [2,3,4]. Fractures of the distal radius (17.5%), metacarpal bones (11.7%), proximal femur (11.6%), ankle (9.0%), and metatarsal bones (6.8%) make up almost 60% of all fractures according to Court-Brown and Caesar’s publication [4]. Although proximal femur fractures are almost always surgically treated, conservative treatment plays an important role in the care of most other common fractures [5,6,7,8].

The basic principles of fracture management were introduced in the beginning of the 20th century: the fracture needs to be reduced, then this must be followed with proper fixation until the broken bone is healed, together with physiotherapy of the free joints (reposition, retention, and rehabilitation). The immobilization of the fracture not only prevents the loss of the reposition or the displacement of the non-dislocated fractures but also protects the area from further injury and effectively relieves the pain. In most unstable, complex fractures, proper fixation can be achieved only with operative treatment (internal fixation); however, non-displaced or stable fractures can be effectively treated with conservative treatment (external fixation). Conservative immobilization can be performed by splinting, casting, bracing, buddy taping, or sling and swathe splint. In acute cases, splinting is the preferred method, because the soft tissue swelling around the injury can cause compression-related injury or strangulation of the extremity. The final fixation of the injured site (usually casting) can be applied between the 5th and 8th day after the trauma [9,10,11,12,13].

Since the introduction of the plaster casting (gypsum plaster or plaster of Paris) in the 10th century, the technique has not significantly changed [14,15,16,17,18]. The advantages of the conventional casts are its easy handling, subsequent plasticity, and low cost, although its heavy weight, low breathability, and lack of water resistance, in addition to the inability to directly observe soft tissue and the possible skin reactions, limit its use. The introduction of fiberglass casts in the 1970s provided a more durable and water-repellent alternative, although they could not eliminate the other disadvantages.

The 3D-printed casts benefit from a custom fit, breathability, lighter weight, and water-proofness, and can also be designed to contain an opening over the wound to avoid pressure points. In addition, they can have an appealing and custom-tailored aesthetic design. These factors can lead to better patient satisfaction and better patient compliance [19,20,21,22].

Graham et al. compared the functionality and patient satisfaction of a fiberglass and 3D-printed short arm cast. They found similar objective functions, but the 3D-printed cast proved to be superior in terms of satisfaction, comfort, and perceived function [14]. In the cited study, the authors did not indicate the 3D-printing technology and material they used but mentioned that these were FDA-approved. In the study of Chen et al., the patients expressed a strong preference for the 3D-printed short arm cast. They produced casts using FDA-approved medically compatible materials with selective-laser sintering or stereolithography 3D printing [23].

Hoogervorst et al. examined the biomechanical characteristic of fiberglass and 3D-printed short arm cast with cadaveric fracture models. They found only a significant difference in the three-point bending test, although this difference was not clinically significant [15]. In their study, HP PA12 nylon material was used, with a HP MultiJet™ 3D printer. Shai et al. used a photopolymer-based DLP additive manufacturing technology. Chen et al. found that the 3D-printed short arm cast could resist appropriate mechanical loads using finite element model analysis and, on the basis of the results, they fabricated a cast using polyamide as a material with selective-laser sintering 3D printing [22].

The main disadvantages of the 3D-printing methods are that they are time-consuming and require special knowledge and instrumentation. The entire procedure could last 6–8 h, but recent developments have significantly accelerated the production. A study by Factor et al. reported an average 161 min workflow for short arm casts using rapid 3D scanning and a DLP high-speed printer operated with liquid photopolymers [16]. A recent review has highlighted that, for the fabrication of upper-limb splints and casts, FFF and SLS technologies are those most frequently used, utilizing thermoplastic polyurethane, ABS, PLA, polypropylene, and polyamide materials [2].

Although there are more studies about clinical applicability and patient satisfaction regarding the 3D-printed casts—and they mainly conclude a good usability—we have found only limited literature regarding the mechanical characterization of the materials used: one short communication examining the mechanical properties of the polymers used for 3D printing and the conventional casting material [24], where several polymers were tested but with only a limited set of mechanical tests. In this study, the results of a three-point bending test showed the flexural and shear strength of PLA to be three times greater compared with a conventional cast.

Some studies examined the mechanical properties of the prototype of the cast, orthosis, or splint created via additive manufacturing. Cazon et al. fabricated an AM splint using VeroWhitePlus™ and TangoPlus™ as model materials and Fullcure 705 ™ as a support material, using PolyJet™ technology. They used a tensile tester to reproduce the four wrist movements (ulnar and radial deviation, flexion, and extension) and compared the tensile strength of a 3D-printed orthosis and a custom-made, low-temperature thermoplastic orthosis. Based on physical tests and finite element analysis, the 3D-printed orthosis was more rigid [25]. Gróski et al. manufactured wrist–hand orthoses with a Raise 3D Pro machine using PLA, ABS, nylon, and high-impact polystyrene materials. Orthoses were measured with a quasi-three-point bending test until the construction cracked or visibly deformed. In terms of material, PLA was the strongest and nylon was the second strongest [26]. In another study, PLA-based specimens and a wrist brace were fabricated with an FDM 3D printer, and a tensile test and Izod impact test were conducted to measure the mechanical properties of the material. The measured values were used as input parameters in a finite-element model to investigate the stresses and displacements under wrist movements [27].

**Figure 1:**
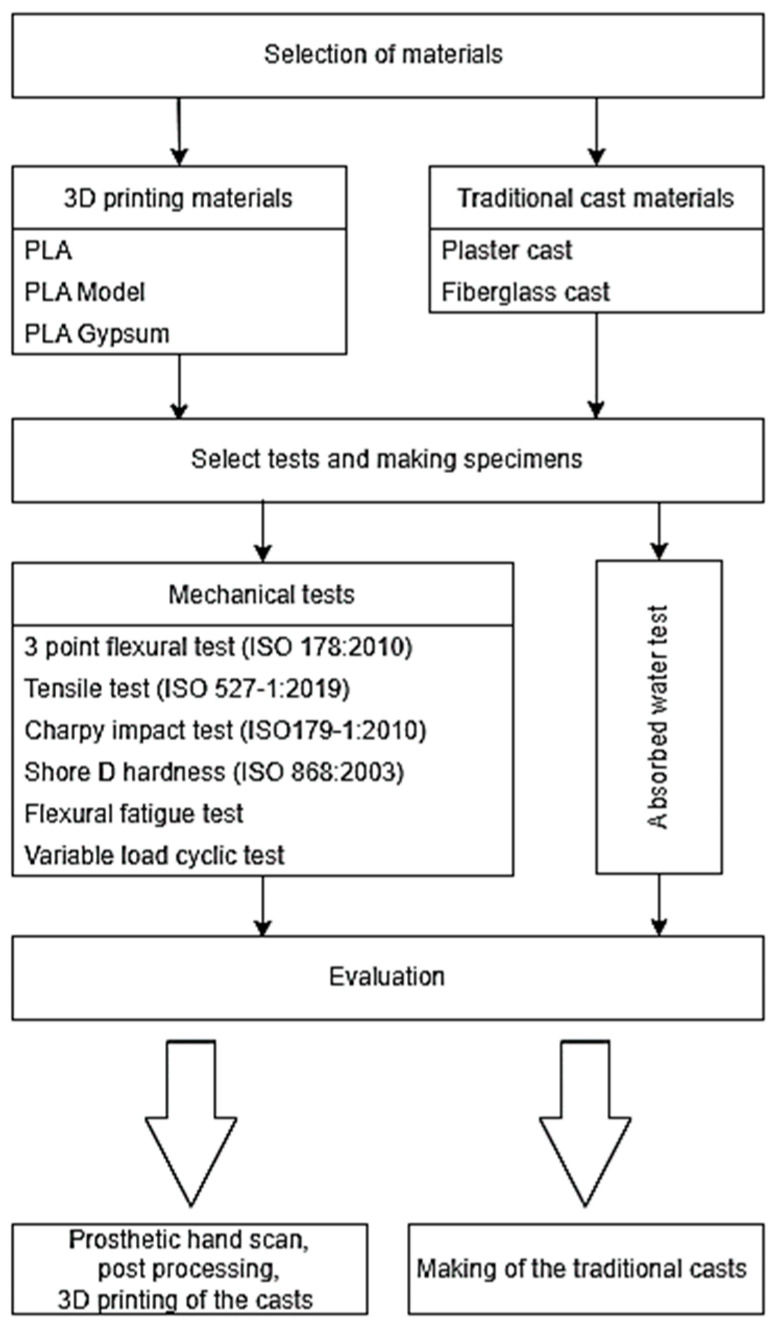
The study protocol.

The rapid development of the 3D-printing technologies (the decrease in the production cost and time, as well as the appearance of almost fully automated processes which do not require specially trained operators) prognosticates these techniques’ fast penetration into the healthcare system, especially for the treatment of fractures. For this reason, we decided to compare traditional and 3D-printed cast materials. Previous studies in the field do not mention gypsum plasters or conventional fiberglass casts as a reference, and dynamic tests are used in only a few studies. Therefore, the aim of our study was to critically evaluate and compare traditional casting methods and materials with devices manufactured with 3D printing using standardized static and dynamic mechanical testing procedures, involving gypsum, fiberglass, PLA, and a promising new PLA–CaCO_3_ composite material [28]. The protocol of the experiments is demonstrated in Figure 1. Additionally, practical aspects such as water-absorption capabilities and cost-effectiveness calculations were considered, which are essential in terms of everyday use, and have not been investigated and compared to date.

## 2. Materials and Methods

We investigated five different materials that could be used for manufacturing casts: three 3D-printable materials and two traditionally applied materials. Among the printable materials, neat PLA and two other PLA-based composites containing calcium carbonate powder were tested: the ‘PLA Model’ contained 20 m/m% CaCO_3_ and ‘PLA Gypsum’ had 45 m/m% CaCO_3,_ according to the manufacturer’s technical data sheet [29].

Among the traditional cast materials, gypsum and fiberglass plaster were examined. To determine the mechanical behavior of the casting materials, static and dynamic mechanical tests were performed, and the mechanical properties of 3D-printed and traditional casting materials were compared. In addition, the water absorption of the specimens was calculated. This was necessary because the lack of water resistance limited the use of plaster, orthosis, or splints. For all measurements, 5-5 specimens were used for each material, except for the flexural fatigue test. A total of 182 specimens were evaluated.

### 2.1. Raw Materials and the Parameters of the Production of Test Specimens

The following two traditional materials were used to prepare test specimens. One was the Safix^®^ Plus plaster cast (Paul Hartmann AG, Heidenheim, Germany). The specimens were cut from the roll with standard sized tools; after that, the layers were dipped in water and placed on a mold, then taken out of the mold and allowed to dry. Another material was the GMed fiberglass cast (Patella-96 kft., Törökbálint, Hungary). It was rolled onto a square-base model according to the protocol, and the samples were cut with a Dremel 8200 cordless multi-tool after drying (Dremel, 1800 W., Mt. Prospect, IL, USA).

**Figure 2:**
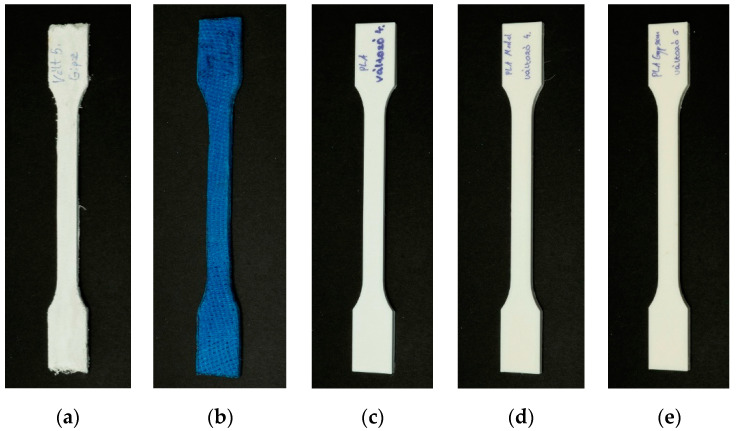
Tensile specimens of the materials (**a**) plaster (gypsum), (**b**) fiberglass, (**c**) PLA, (**d**) PLA Model, and (**e**) PLA Gypsum.

The PLA-based specimens were printed using a Craftbot Plus 3 (Craftbot Ltd., Budapest, Hungary) 3D printer with a 0.4 mm nozzle, 0.2 mm layer height, and 100% infill density. The 215 °C primary extruder temperature and 65 °C heated-bed temperature provided the necessary amount of heat for fabrication. The following materials were printed: Filaticum PLA (Filamania Kft., Szigetszentmiklós, Hungary), Filaticum Gypsum (Filamania Kft., Szigetszentmiklós, Hungary), and Filaticum Model (Filamania Kft., Szigetszentmiklós, Hungary). Both filaments are PLA-based with CaCO_3_ (PLA Model: 20 m/m% CaCO_3_ PLA Gypsum: 45 m/m% CaCO_3_). Each filament’s diameter was 1.75 mm and they had a white color. The specimens were sliced using the CraftWare™ software (Craftbot Ltd., Budapest, Hungary). The specimens are shown in Figure 2.

### 2.2. Mechanical Tests

#### 2.2.1. Three-Point Flexural Test

The 3-point bending tests were carried out using a Zwick/Roell Z100THW universal material tester (ZwickRoell, Ulm, Germany). The tests were based on the ISO 178:2010 standard with the preferred test specimen. The size of the specimen was 4 mm × 10 mm × 80 mm. The pre-load was 0.1 MPa and the testing speed was set to 2 mm/min during the full test. The support distance was 64 mm and the maximal deformation was 4.7% according to the standard.

#### 2.2.2. Tensile Test

The tensile behavior of the specimens was determined using the same Zwick/Roell Z100THW test machine (ZwickRoell, Ulm, Germany) with an extensometer, except in the case of plaster cast specimens. The tests were conducted according to the ISO 527-1:2019 standard with the preferred test specimen. The specimen was an A1 from the ISO 527-2:2012 standard. The pre-load was set to 0.1 MPa; testing speed was 1 mm/min for the determination of the Young’s modulus and was then set to 50 mm/min for the tests.

#### 2.2.3. Charpy Impact Test

To measure the impact values, the specimens were tested using a Zwick/Roell Hit50P (manufacturer: ZwickRoell, 89079, Ulm, Germany) instrument utilizing a 5 J pendulum, following the ISO 179-1:2010 standard. The size of the specimen was 4 mm × 10 mm × 80 mm; the edgewise impact was performed on the test specimen without notch.

#### 2.2.4. Shore D Hardness

The Shore D hardness tester was a Zwick/Roell 3131/320154 (ZwickRoell, 89079, Ulm, Germany). The tests were performed according to the ISO 868:2003 standard. The instrument was set on a stable stand during the entire measurement process. For the fiberglass cast, the hardness was not measurable due to its inhomogeneous, gauze-like mesh structure.

#### 2.2.5. Flexural Fatigue Test

**Figure 3:**
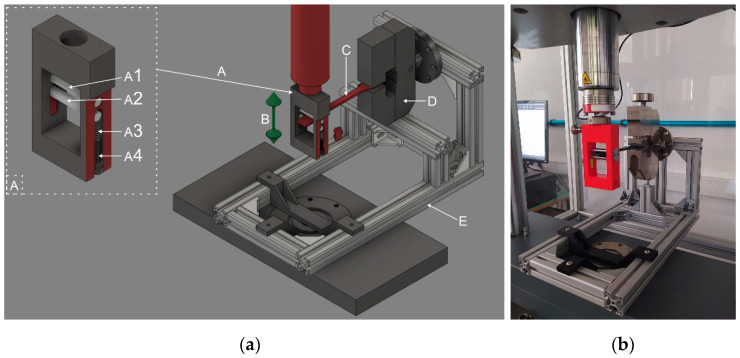
(**a**) The models used in the setup of the flexural fatigue test measurement: (a) special gripper; (a1) fixed roller; (a2) moving roller; (a3) spring; (a4) adjusting screw; (b) moving direction; (c) specimen; (d) screw grip; and € support frame. (**b**) The assembled device for the flexural fatigue test measurement [30].

To investigate the effect of repetitive loading, a flexural fatigue test was performed. The tests were carried out on all specimens using a Zwick/Roell e/m actuator material tester (ZwickRoell, 89079, Ulm, Germany) with a 5-kN load cell. The type of specimen was the A1 from the ISO 527–2:2012 standard. The testing frequency was 2 Hz, and the movement was sinusoidal. The sample gripper and measuring system are shown in Figure 3 and were used in a previous study [30].

Deflection started at 50 mm and decreased by 5 mm with each new test. The measurements were carried out until the specimens broke. The breaking point was determined when the flexural stress was decreased by 50%.

The flexural stress was calculated using the following formula:

(1) $$\sigma =\frac{6\cdot F\cdot l}{a\cdot b^{2}}$$

where *σ* indicates flexural stress, *F* is force, *l* is the test length of the specimen, *a* is the width of the specimen, and *b* is the thickness of the specimen.

The fatigue limit was 50% of the maximum stress.

#### 2.2.6. Variable Load Cyclic Test

To examine the permanent deformation of the materials, a variable load cyclic test was carried out, testing whether a larger load following a smaller one provided the same support. The tests were carried out using the same Zwick/Roell e/m actuator. The specimen type was A1 according to the ISO 527–2:2012 standard.

**Figure 4:**
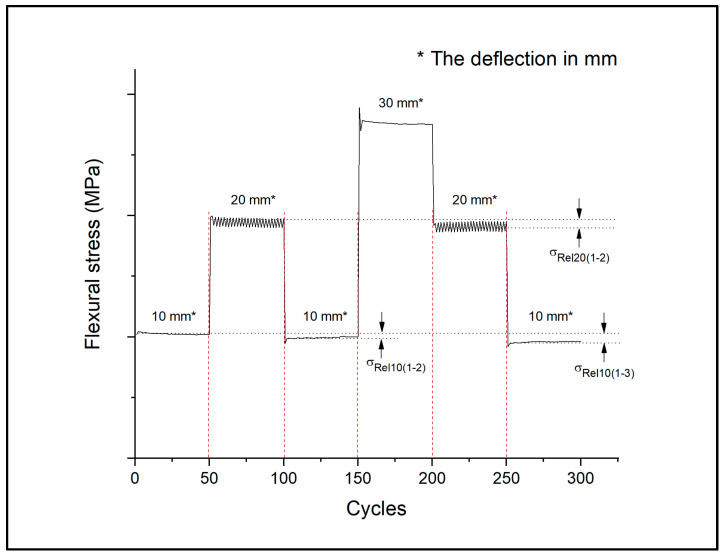
The scheme for variable load cyclic test method. The figure represents how the specimens were bent to a specified value for 50-50 cycles. In the first stage, the deflection was 10 mm, then 20 mm, 10 mm, 30 mm, 20 mm, and 10 mm again. The black dotted lines show flexural stress at 10 mm and 20 mm deflection and the relative changes in stress are illustrated by the arrows.

During the test, the test specimens were subjected to cyclic deflections of 10 mm at first, then 20 mm, 10 mm, 30 mm, 20 mm and, finally, 10 mm again for 50-50 cycles; the testing frequency was 1 Hz. The measured and calculated values can be seen in Figure 4.

### 2.3. Absorbed Water Content Measurement

All the specimens were placed in distilled water at room temperature (24 °C) for 24 h. The samples were fully surrounded/covered by water on all sides. The mass of the dry specimens was measured by an analytical balance (Ohaus Discovery DV215CD—Ohaus Corporation, 7 Campus Drive, Suite 310, Parsippany, NJ 07054, USA). After 24 h of soaking, the specimens were carefully wiped and weighed again. Each sample type contained 5 specimens of the same shape and size.

### 2.4. Digital Microscopy

The surfaces of the casts were compared using a König CMP-USBMICRO30 Digital microscope (König Electronic GmbH, 64385 Reichelsheim, Germany) with 50× magnification.

### 2.5. Manufacturing of the Traditional and 3D-Printed Casts

The first step in conservative fracture treatment is the repositioning of the fracture, when necessary: the positioning of the involved joints and the securing of this stance. Before a traditional cast is applied, the affected area should be covered with a soft material liner to protect the skin and pressure points. The next step is to measure the required length of the splint and/or the optimum width of the plaster cast roll. This is followed by the immersion of the casting material into water, along with the process of squeezing the excess water. The casting material is applied to the affected area, usually from distal to proximal with overlapping rolls. Before applying the last layer, the ends of the lining should be folded back to avoid sharp edges. After application, it is necessary to smooth the surface and check for sharp edges or points. Once the position is fixed, the material must be hardened (usually 15–45 min).

**Figure 5:**
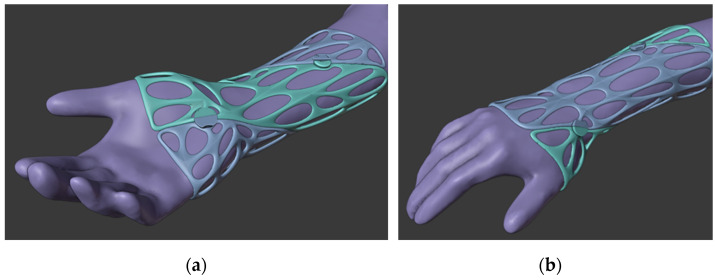
Three-dimensional models of the braces in Blender (**a**) palmar view of the forearm and (**b**) dorsal view of the forearm.

To create 3D-printed fracture fixation components, a surface model of the patient’s limb must first be created; contact-free active 3D scanning is the ideal solution for this. A forearm prosthesis Ottobock 8S4 = 206 × 85R (Ottobock SE & Co. 37115 Duderstadt Germany) was used as a model for the study for both conventional casting and 3D scanning. For the latter, scans were taken using a Sense 2 handheld scanner (manufacturer: 3D Systems, Rock Hill, SC, USA). The raw spatial mesh model of the forearm and wrist was first cleaned and optimized in the mesh modeling software Blender 3.0 (Blender Foundation, Buikslotermeerplein 161, 1025 ET Amsterdam, The Netherlands). As the next step, on the basis of the skin surfaces that were directly covered by the fixture, a Voronoi patterned orthosis was created by 3D modelling (Figure 5). For a more accurate fit and stiffer grip, the orthosis elements were given a spiral design. To provide the necessary grip on the forearm, fixation clamps were placed on the model, which could be secured together with o-rings. The clamps could be released so that the brace could be removed or replaced as required. The finished model was physically produced on a Craftbot 2 XL (Craftbot Ltd., Salgótarjáni út. 12-14., Budapest, Hungary) 3D printer.

### 2.6. Statistics and Analysis

The values of mechanical tests were compared using one-way ANOVA and Tukey post-hoc tests. A curve was fitted to the data measured from the flexural fatigue tests using the following formula:

(2) $$y=y_{0}+A_{1}e^{-\frac{x}{t_{1}}}+A_{2}e^{-\frac{x}{t_{2}}}$$

where *y*_0_ is the limit value of function and *A*_1_, *A*_2_, *t*_1_, and *t*_2_ are constants.

The statistical analysis and curve-fitting were carried out using the Origin 2018 software (OriginLab Corporation, One Roundhouse Plaza, Northampton, MA, USA).

For variable load tests, the maximum deflection stress of the cycles was determined using the peek analyzer in OriginLab, and the relative changes in flexural stress (*σ*_Rel_) were calculated by comparing the values measured at the first 10 mm and 20 mm deflection intervals.

## 3. Results

### 3.1. Tensile and Flexural Test

The results of tensile and flexural tests are presented and discussed so that their main parameters are easier to compare.

**Figure 6:**
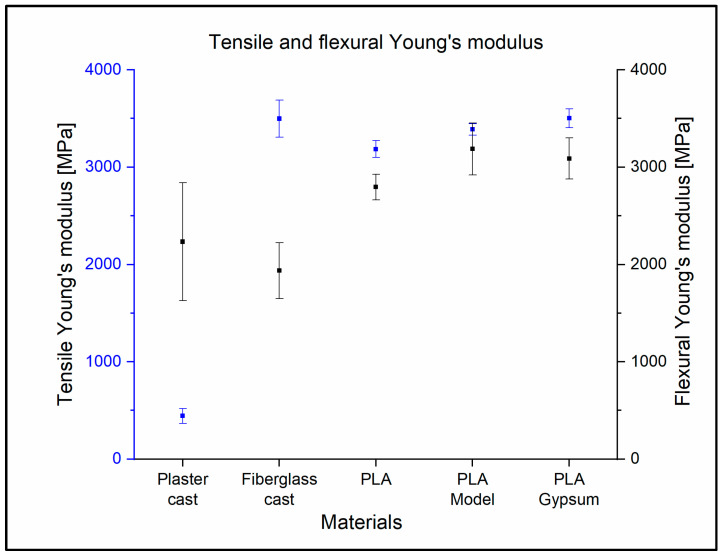
Tensile and flexural Young’s modulus of the casting materials. Black squares indicate the mean of the flexural Young’s modulus; black lines refer to standard deviations in case of flexural test. The blue squares and lines indicate the mean of tensile Young’s modulus and standard deviation in tensile tests.

The tensile Young’s modulus of the plaster cast was the smallest: 443 MPa ± 75.0 MPa. The fiberglass cast value was 3498 MPa ± 192 MPa. The Young’s modulus of the 3D-printed materials was between 3186 MPa ± 85.5 MPa (PLA) and 3502 ± 97.1 (PLA Gypsum) (Figure 6). The value of the ANOVA test is *p* < 0.0001. These results apply for all mechanical tests, so they will not be presented in more detail. For the Tukey post-hoc test, the fiberglass cast, the PLA Model, and the PLA Gypsum did not show any significant differences from each other. The results of all the ANOVA and Tukey post-hoc tests can be found in the Supplementary Material: Tables.xlsx; mechanical tests sheet. The biggest relative difference between the fiberglass cast and 3D-printed materials is 9.7%.

During the flexural tests, the plaster cast’s Young’s modulus was 2233 MPa ± 605 MPa. The fiberglass cast was found to be the most flexible material (1936 MPa ± 289 MPa). The most rigid material was the PLA Model; the measured data were 3186 MPa ± 265 Mpa, in this case (Figure 6). When comparing the traditional casts and 3D-printed materials using Tukey tests, only the fiberglass cast and the PLA showed no significant difference.

**Figure 7:**
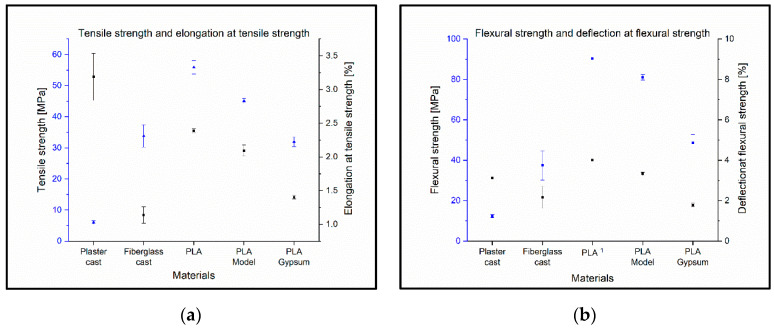
(**a**) Tensile strength and elongation at tensile strength with standard deviations. Blue squares and lines indicate the mean and standard deviation of the tensile strength; black squares show the value of elongation with standard deviation at tensile strength. (**b**) Flexural strength and deflection of flexural strength. The mean of flexural strength with the standard deviation is indicated by the blue squares and line, and the black squares and line refer to the deflection and its standard deviation at flexural strength. ^1^ In the case of PLA material, only one specimen was broken during the tests.

The tensile strength of the plaster cast was the lowest, at 6.04 MPa ± 0.53 MPa. The value of the fiberglass cast was measured as 33.8 MPa ± 3.6 MPa. The highest value was found for the PLA, with 55.9 MPa ± 2.15 MPa. Only the fiberglass cast and the PLA Gypsum showed no significant difference in the Tukey tests (Figure 7a).

Surprisingly, the elongation at the tensile strength of the polymer-based raw materials (including the 3D-printed and fiberglass cast materials) was proportional to the tensile strength, although the plaster cast had the highest elongation at the tensile strength *ε*_M_ = 3.2%. The smallest elongation was observed for the fiberglass cast, with a value of 1.1% (Figure 7a).

The plaster cast had the lowest flexural strength, with a value of 12.3 MPa ± 0.8 MPa, the fiberglass cast was 37.4 MPa ± 7.1 MPa, and the highest value was obtained for the PLA model, with 81.1 MPa ± 1.4 MPa. PLA was not considered (Figure 7b) because only one measurement was relevant. During the bending tests, four pieces of PLA specimen did not break; therefore, only one flexural strength value was available for this material. The flexural stress at a standard deflection (3.5%) of the PLA material was 90.5 MPa ± 1.64 MPa.

Interestingly, fiberglass cast had the smallest deformation in flexural strength, *ε*_fM_ = 1.8%. In the case of plastic-based materials, the deflection rate for flexural strength decreases with elevations in calcium carbonate content (Figure 7b).

### 3.2. Charpy Impact Test

To test the impact resistance, Charpy impact tests were performed, and the results are shown in Table 1. For PLA-based materials, the impact strength was affected by the calcium carbonate content, but PLA Gypsum also had a higher impact resistance than a conventional gypsum plaster cast. The highest impact strength was observed for the fiberglass cast, with a value of 22.49 kJ/m^2^ ± 4.09 kJ/m^2^, but the standard deviation was also higher compared to the other materials. In all cases, the materials showed significant differences in the Tukey test.

### 3.3. Shore D Hardness

In the fiberglass cast, the hardness could not be measured because its structure was not homogenous but meshed. The hardest material in the tests was the PLA; in this case, Shore D hardness was 76.64 ± 0.95, and the least hard material was plaster cast, with a result of 64.44 ± 5.25. Further results can be found in Table 2. The materials showed significant differences in the Tukey test, except for the PLA-PLA Model.

### 3.4. Flexural Fatigue Test

**Figure 8:**
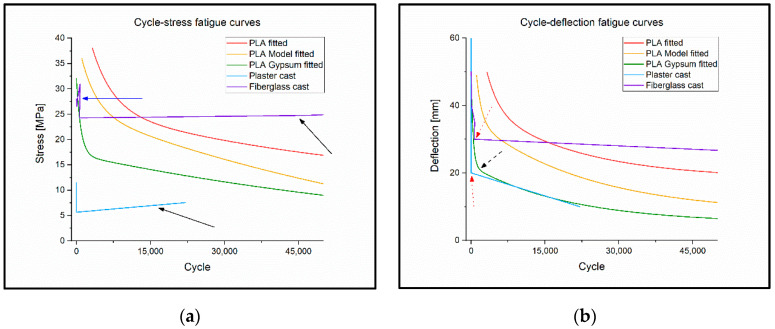
(**a**) Cycle–stress fatigue curves. The black arrows shows that plaster cast and fiberglass cast do not follow the decreasing trend. The blue arrow shows that the fiberglass swings above 26 MPa stress. (**b**) Cycle–deflection fatigue curves. The red dotted arrows on the diagram show that the plaster cast and the fiberglass cast did not follow the classic s–n curve and also have a break point in the function. The black dashed arrow indicates that the function fitted to the measured values of PLA Gypsum with higher gypsum content suddenly changes.

During the flexural fatigue test, the PLA, PLA Model, and PLA Gypsum showed a normal fatigue curve in the stress–cycle diagram. However, for the PLA Gypsum, a sudden change was observed in the fitted function at 17 MPa (Figure 8a). In the deflection–cycle diagram, a sudden change in the graph occurred at approximately 20 mm deflection, as shown in Figure 8b.

For the plaster cast, if the deflection was more than 10 mm, then the flexural stress decreased under the fracture limit in the second cycle.

Fiberglass cast fatigue also could not be detected. As the stress went above ~26 MPa, the values began jumping back and forth without any trends. Below this value, this specimen’s load capacity significantly increased with time. At 24.8 MPa, the cycle number of the fracture was the 45,949th. The fracture of the curve is visible at 30 mm on the fitted deflection–cycle diagram (Figure 8b). The deflection–cycle values of the fiberglass cast can be seen in Table 3.

At 20 mm deflection, fatigue fracture of the PLA specimen occurred when the load reached 16.7 MPa, and the test specimen constructed from a PLA Model broke at 19 MPa load. At lower loads, the specimens suffered permanent deformations but they did not break. Test specimens of PLA Gypsum broke at a deflection of 10 mm when the load was 12 MPa.

### 3.5. Variable Load Cyclic Test

The results show that the materials did not have a breaking point during the fatigue tests and were able to exert the same resistance during the subsequent test cycles as they did with the previous deflection of the same amount.

The plaster cast showed the most significant change; when the same amount of bending was repeated, the holding force was greatly reduced. Over the course of the first 10 mm bending interval, the value of the measured stress was 2.66 MPa ± 0.63 MPa; in the second 10 mm deflection stage, the relative change was −59.0%; in the third stage, *σ*_Rel10(1–3)_ = −79.7%. At 20 mm deflection, the relative change was −52.4% for the plaster cast. PLA showed the lowest relative change in the second deflection stage: *σ*_Rel10(1–2)_ = −2.2% and *σ*_Rel20(1–2)_ = −2.1%; and in the third stage, *σ*_Rel10(1–3)_ = −4.8%. All measured values of the variable load cyclic tests can be found in the Supplementary Material: Tables.xlsx; variable load test sheet.

**Figure 9:**
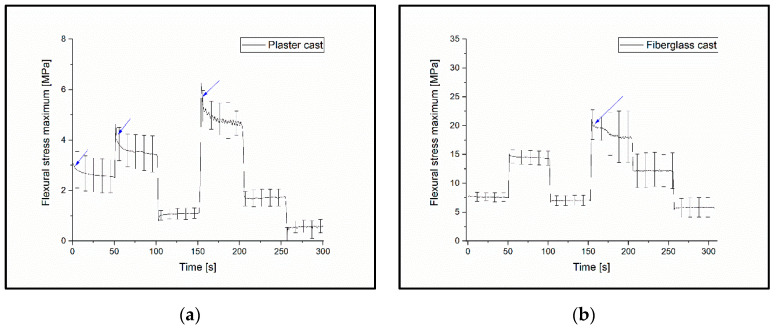

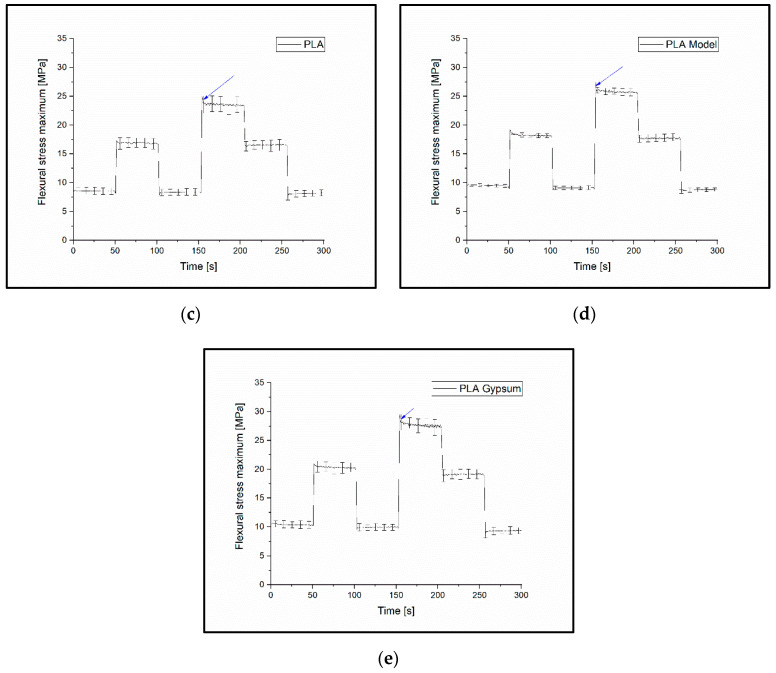
Measured values of the variable load cyclic tests. The blue arrows show the points at which permanent changes have occurred in the materials: (**a**) plaster cast, (**b**) fiberglass cast, (**c**) PLA, (**d**) PLA Model, and (**e**) PLA Gypsum (the results show the materials that did not have a break point on the curves in the fatigue test; these were able to reproduce the previous holding force after a higher load under the same deflection).

The flexural stress maximum decreased in all cases, as illustrated in Figure 9.

### 3.6. Absorbed Water Content Measurement

**Figure 10:**
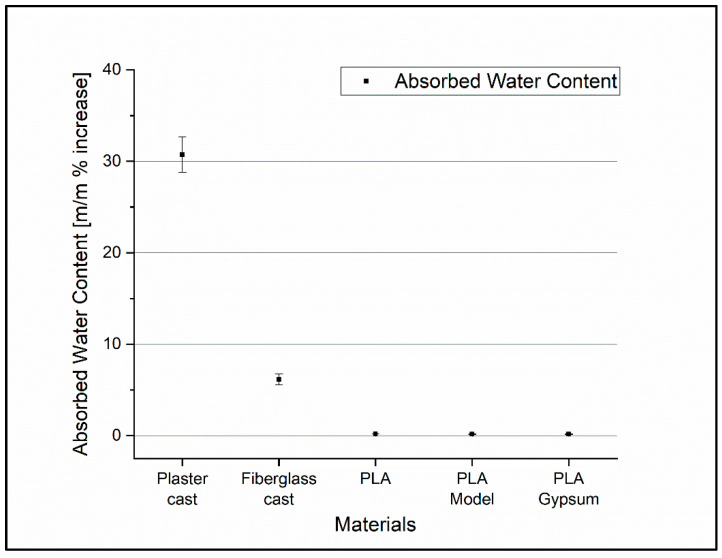
The change in mass of examined materials in percentage and their standard deviations. Percentage change and standard deviation of the mass of the test substances.

The amount of water absorbed by different cast and 3D-printed samples was calculated to determine the water-repellency of the materials. The amount of water that was absorbed is represented as a percentage increase in the mass of the specimen (m/m %). The errors are standard deviances calculated from five independent measurements. Paired *t*-tests were performed for the dry and wet mass of the specimens and the difference was extremely statistically significant, with *p* < 0.0001 in all cases (Figure 10). However, this very significant change is due to the low variance in some cases. The largest relative change for 3D-printed materials was 0.19%.

### 3.7. Traditional and 3D-Printed Casts

The medical time for the Sofix^®^ plaster cast is 0.75 h, during which it sets; after 72 h, it can be fully loaded. The GMed fiberglass cast’s binding time ranges from 3 min to 5 min; it can be loaded after 30 min, although the full drying time is 24 h.

The entire workflow of the printed braces took approximately 26.3 h to complete, from the start of scanning the subject to the installation of the braces. The time and materials needed to prepare the casts are given in Table 4.

**Figure 11:**
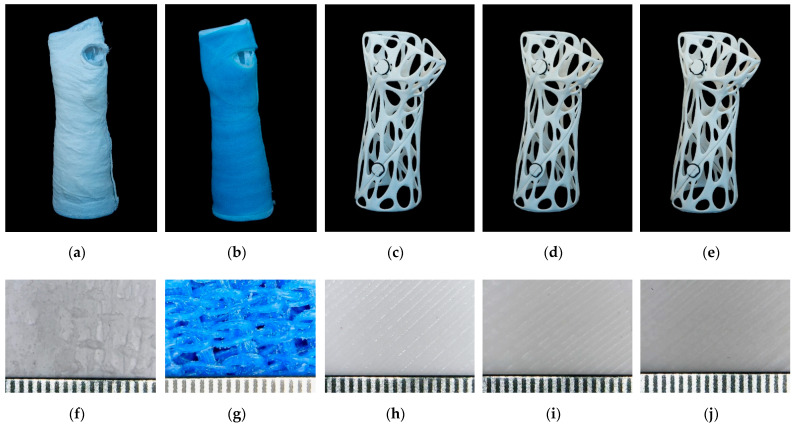
Photos of the casts (**a**–**e**): (**a**) plaster cast; (**b**) fiberglass cast; (**c**) PLA; (**d**) PLA Model; (**e**) PLA Gypsum; (**f**–**j**) video microscopic image of the surfaces of the casts, with a ruler with mm scale at the bottom of the pictures; (**f**) plaster cast’s surface, where the white gypsum shows the gauze fibers that stiffen the casting; (**g**) fiberglass cast’s surface, showing a woven model of the fiberglass cast; (**h**) PLA’s surface; (**i**) PLA Model’s surface; (**j**) PLA Gypsum’s surface; (**h**–**j**) the surface is typical for the FFF 3D-printed thermoplastic materials, and the striping is due to extrusion.

A digital microscopic examination of the surface of the materials showed the following: the white gypsum particles and the gauze fibers that stiffen the casts are visible on the surface of the plaster casts and have an inhomogeneous structure (Figure 11f). The woven structure of fiberglass is also visible on the surface (Figure 11g). In the case of PLA and the composites, the surface is typical for the thermoplastic FFF 3D-printed materials. The observed striping is due to the extrusion process (Figure 11h–j).

## 4. Discussion

Additive manufacturing is a promising technology that could be used to develop customized, more convenient, lightweight forearm braces and casts; however, it requires the correct choice of materials and processes.

Although there are more studies about the clinical suitability of 3D-printed casts, splints, and orthoses [14,15,16,19,20], limited information is available on the mechanical properties of the materials; additionally, a critical comparison with traditional plaster and fiberglass casts is missing. Therefore, in this study, the mechanical characteristics of traditional casting materials and some promising 3D-printable materials, including PLA and PLA–CaCO_3_ composites, were investigated and compared by studying the two types of manufacturing processes.

The tensile Young’s modulus of the fiberglass cast is very similar to the values measured in the case of 3D-printed materials. This is no longer the case for the Young’s modulus in bending. The fiberglass cast is much more elastic in terms of bending than tension, with a Young’s modulus of 3498 MPa ± 192 MPa and 1936 MPa ± 289 MPa, i.e., the values are almost halved. The plaster cast, on the other hand, significantly increased by about five times, with a tensile elasticity of 443 MPa ± 75 MPa but a flexural elasticity of 2233 MPa ± 606 MPa. This is due to the inhomogeneous mesh structure of the material.

The 3D-printed materials were shown to have a higher tensile strength and flexural strength than plaster cast and fiberglass cast. This opens up the possibility that the cast from 3D-printed materials could be thinner or have a more open structure. This underlines and clearly demonstrates that lightweight and breathable structures can be fabricated with FFF 3D-printing without a decrease in tensile strength and flexural strength, which is a favorable property in terms of clinical applications. Qin et al. measured neat PLA specimens and PLA specimens with different concentrations of CaCO_3_, where the tensile strength of neat PLA was 54.7 MPa and the PLA with 20% CaCO_3_ was 49 MPa. In this study, 55.9 MPa ± 2.1 MPa was measured for neat PLA and 45.1 MPa ± 0.8 MPa for PLA with 20% CaCO_3_ content. This indicates that the expected tensile strength decreases with increasing CaCO_3_ concentration [31].

The plaster cast showed a low impact strength of 4.9 kJ/m^2^ ± 0.4 kJ/m^2^ with a scattering of almost 10%, due to the casting process. The fiberglass cast best resisted the dynamic forces at 22.5 kJ/m^2^ ± 2.8 kJ/m^2^, although they showed a rather large standard deviation. This may be due to the mesh structure of the material and the fact that it was rolled onto a test specimen according to hospital protocol, thus increasing its inhomogeneity. Of the 3D-printed materials, PLA showed the highest value of 15.6 kJ/m^2^ ± 0.5 kJ/m^2^; this was reduced by the addition of calcium carbonate to 8.0 kJ/m^2^ ± 0.2 kJ/m^2^ (PLA Gypsum). These findings highlight that the polymer- and composite-based casts have a significantly greater resistance to dynamic forces, which is important if a patient falls or bumps the injured and casted arm on an object.

The plaster cast and the fiberglass cast did not show normal fatigue; the reason for this is likely due to its structural and material properties, such as its inhomogeneous, mesh-like structure and, in the case of the plaster cast, the presence of gauze fibers. The fatigue curves of the 3D-printed materials show that there is an increasing, sudden change in the function as the gypsum concentration increases, so it is not recommended to print fixings with a higher gypsum concentration than the PLA Gypsum filament. Such tests have been carried out only on PLA to date, the results of which are the same as the current ones [30].

The alternating load on the casts and its effect on the permanent deformation of the device was demonstrated by a variable load cyclic test. The relative change in flexural stress showed the maximum value at the same amount of deflection, reaching significance. A greater than 50% decrease was observed for the plaster cast if the same amount of load was used before and after a higher load. The relative change in the case of the 3D-printable materials was markedly lower; however, it increased with the CaCO_3_ content. These findings and fatigue test results suggest that PLA and the CaCO_3_ composites are more likely to retain or regain their shape after a higher load compared to traditional cast materials. The usual fixation period for a fracture varies between 4 and 12 weeks; therefore, the final casting must endure weeks of continuous loading (reflex movements, movements during sleeping, accidental impacts, etc.). The cast material which can better resist this fatigue needs to be changed less frequently (or not at all), imposing a lower load on the healthcare system and reducing expenses. In addition, the traditional cast materials, which do not regain their original shape after loading, carry the threat of the patient’s developing bony malalignment.

Regarding the absorbed water content measurements, the fiberglass cast, known as a water-repellent, traditional cast material showed a more than 5 m/m% increase in the mass of the specimen after 24 h of soaking. In contrast, the 3D-printable cast materials did not absorb substantial amounts of water; the largest relative change was 0.19%. Wahit et al. reported similar findings in the case of the PLA. Due to the low levels of water absorption, the rigidity of the device was maintained which allows for efficient fixation. This also offers the possibility of an easier cleaning process and improves patient hygiene [32]. The comfort can be also higher for 3D-printed materials because it did not absorb a significant amount of sweat, thus preventing bad odors.

Manufacturing a 3D-printed cast is a time-consuming process. In this work, the entire procedure took 26.3 h; however, based on previous studies, the total time ranged from approximately 2.7 h to 3–5 days depending on the brace structure, material, and the 3D-printing technology [16,33]. It should be noted that the printing time greatly depends on the size of the hand, the selected geometry, the materials used, and the printing parameters. Traditional methods are clearly less time-consuming and more cost-efficient at present, but the trends and international research works clearly show a decrease in printing time and costs in recent years. It should be noted that the price of fiberglass casts and 3D-printed casts are comparable (fiberglass: EUR 16.8 per piece, 3D-printed materials: between EUR 6.8 and EUR 18.3 per piece). Another advantage of 3D-printing technology is that it has no binding time, so it can be loaded immediately after application and can be removed and reinserted for control imaging studies (avoiding the image-quality-worsening effect of the traditional casts). Therefore, additive manufacturing could be a good alternative for customized splints and casts in the near future.

## 5. Conclusions

The present study reveals that FFF 3D-printed materials such as PLA and PLA–CaCO_3_ composites generally have better mechanical properties and water-absorption characteristics compared to traditional plaster of Paris or fiberglass materials. Regarding these results, it is important to highlight the observations related to the variable load tests. These demonstrated that the 3D-printed casts do not necessarily need to be changed after an impact or a higher load, because the PLA and CaCO_3_ can reproduce their original shape. These features are beneficial for the patients and, in the long-term, for the healthcare system, leading to improved patient outcomes and better patient adherence and cooperation, as well as a lower number of complications, such as infections or repeated injuries. Additionally, it is demonstrated that the costs of the 3D design and fabrication processes have significantly decreased in recent years, and they have become less dependent on technology. However, at present, the prices and time needed for application are still more favorable for traditional methods.

## Figures and Tables

**Table 1:** The measured Charpy impact strength values.

Material	Charpy Impact [kJ/m^2^]	SD [kJ/m^2^]
Plaster cast	4.87	0.20
Fiberglass cast	22.49	4.09
PLA	15.56	0.19
PLA Model	13.07	0.28
PLA Gypsum	7.98	0.09

**Table 2:** Shore D hardness measure values.

Materials	Shore D Hardness	SD
Plaster cast	64.44	5.25
Fiberglass cast ^1^	---	---
PLA	76.64	0.95
PLA Model	75.58	0.36
PLA Gypsum	69.56	1.51

^1^ Shore D harness could not be measured for the fiberglass cast due to the inhomogeneous mesh structure.

**Table 3:** The deflection–cycle values of fiberglass cast.

Deflection [mm]	Cycle Number at Break
25	75,649
27	45,949
30	646
35	727
40	67
45	55
50	15

**Table 4:** Summarizing the material usage and the labor time.

	Weight of Material Used without/with Support [g/Casts]	Material Cost [EUR/Cast] ^1^	Medical Hours with the Patient [h]	Modelling [h]	Printing Time [h]	Support Removal and Installation [h]	Total Time [h]
Plaster cast	457/--- ^2^	3.1	0.75	---	---	---	0.75
Fiberglass cast	325/---	16.8					
PLA	318/400	6.2					
PLA Model	330/410	17.0	0.5	1.5	23.8	0.5	26.3
PLA Gypsum	346/440	18.3					

^1^ Based on HUF to EUR conversion on 18 July 2022. (404 HUF/EUR). ^2^ Weight of plaster cast 24 h after application.

## Data Availability

The data presented in this study are openly available at Mendeley Data: (Schlegl, Adam; Zoltán, Ujfalusi; Kardos, Kinga; Szőke, Andras; Maroti, Peter; Told, Roland (2022), “Evaluation and comparison of traditional plaster and fiber-glass casts with 3D-printed PLA and PLA-CaCO_3_ composite splints for bone fracture management”, Mendeley Data, V2, doi: 10.17632/5wfvwsb3x3.2).

## Supplementary Materials

All the data presented in this study are openly available at Mendeley Data. DOI: 10.17632/5wfvwsb3x3.1 (Tables.xlsx; containing all data measured regarding the mechanical test).
